# Improving the viability of tissue‐resident stem cells using an organ‐preservation solution

**DOI:** 10.1002/2211-5463.12748

**Published:** 2019-11-18

**Authors:** Takaya Suzuki, Chiharu Ota, Naoya Fujino, Yukiko Tando, Satoshi Suzuki, Mitsuhiro Yamada, Takashi Kondo, Yoshinori Okada, Hiroshi Kubo

**Affiliations:** ^1^ Department of Thoracic Surgery Institute of Development, Aging and Cancer Tohoku University Sendai Japan; ^2^ Department of Pediatrics Tohoku University Hospital Sendai Japan; ^3^ Department of Respiratory Medicine Tohoku University Graduate School of Medicine Sendai Japan; ^4^ Cell Resource Center for Biomedical Research Institute of Development, Aging and Cancer Tohoku University Sendai Japan; ^5^ Department of Thoracic Surgery Japanese Red Cross Ishinomaki Hospital Ishinomaki Japan; ^6^ Department of Advanced Preventive Medicine for Infectious Disease Tohoku University Graduate School of Medicine Sendai Japan

**Keywords:** mesenchymal stem cell, organ preservation, preservation solution, progenitor cell, stem cell, tissue transport

## Abstract

Human clinical specimens are a valuable source of tissue‐resident stem cells, but such cells need to be collected immediately after tissue collection. To extend the timescale for collection from fresh human samples, we developed a new extracellular fluid (ECF)‐type preservation solution based on a high‐sodium and low‐potassium solution containing low‐molecular‐weight dextran and glucose, which is used for preservation of organs for transplantation. In this study, we compared the preservation of tissue‐resident stem cells using our ECF solution with that using three other solutions: PBS, Dulbecco’s modified Eagle’s medium and Euro‐Collins solution. These solutions represent a common buffer, a common culture medium and a benchmark organ‐preservation solution, respectively. Lung tissues were removed from mice and preserved for 72 h under low‐temperature conditions. Of the solutions tested, only preservation in the ECF‐type solution could maintain the proliferation and differentiation capacity of mouse lung tissue‐resident stem cells. In addition, the ECF solution could preserve the viability and proliferation of human alveolar epithelial progenitor cells when stored for more than 7 days at 4 °C. The mean viability of human alveolar type II cells at 2, 5, 8 and 14 days of low‐temperature preservation was 90.9%, 84.8%, 85.7% and 66.3%, respectively, with no significant differences up to 8 days. Overall, our findings show that use of our ECF‐type preservation solution may maintain the viability and function of tissue‐resident stem cells. Use of this preservation solution may facilitate the investigation of currently unobtainable human tissue specimens for human stem cell biology.

## Abstract 2

Here, we describe a newly developed extracellular fluid‐type organ preservation solution that maintained the viability of human lung stem/progenitor cells, such as alveolar type II cells, during 7‐day refrigerated preservation after the collection of lung specimens in local hospitals. This ready‐to‐use solution may be suitable for the transport of human clinical specimens from hospitals to scientific and bioengineering laboratories.

## Graphical abstract

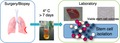

**Abbreviations**
ATII: alveolar type II
CD: cluster of differentiation
DMEM: Dulbecco’s modified Eagle’s medium
ECF: extracellular fluid
EpCAM: epithelial cell adhesion molecule
FABP: fatty acid–binding protein 4
FACS: fluorescence‐activated cell sorting
FITC: fluorescein isothiocyanate
ICF: intracellular fluid
MEF: mouse embryonic fibroblast
PE: phycoerythrin
PI: propidium iodide
Sca‐1: stem cell antigen‐1
SP‐B: surfactant protein B
SP‐C: surfactant protein C

Adult organs contain tissue‐resident stem or progenitor cells that are responsible for organ homeostasis and repair after injury 1. Because of their ability to continuously replenish mature adult cells throughout life, tissue‐resident stem cells are considered to play an important role in organ development, aging and tumorigenesis 1, 2. Tissue‐resident stem cells are also a potential source of cells for cell‐based therapies, disease modeling and drug screening 3, 4. In addition to tissue‐resident stem cells, adult cells originating from patients would be ideally applicable for creating artificial stem or progenitor cells 5, 6 and for the production of bioengineered organs 7. As such, there is an increasing demand for human clinical samples as sources of adult cells.

Biopsy samples or surgical specimens should be processed immediately after tissue collection to isolate viable cells from adult organs 8. Such collection, however, often poses a great effort for investigators. It stands to reason then that if specimens from humans could be kept viable for several days, clinical samples that are currently unattainable in basic research laboratories would be available for experimentation. Furthermore, investigators would be able to evaluate and use clinical tissue samples from distant locations, including overseas hospitals. However, despite its importance and demand, little attention has been given to the challenge of preserving stem cell viability during tissue transportation 9.

In the field of organ transplantation, several types of organ‐preservation solutions have been developed to maintain the viability and function of donor organs. These preservation solutions are classified into two groups: intracellular fluid (ICF)‐type preservation solutions (high potassium/low sodium ratio) and extracellular fluid (ECF)‐type preservation solutions (low potassium/high sodium ratio) 10. Earlier investigations exploring the use of preservation solutions for transplantation have usually employed ICF‐type solutions 10; however, more recent studies have suggested the improved functionality of the organs preserved in ECF‐type solutions 11, 12, 13, 14. We previously demonstrated the improved viability and function of alveolar type II (ATII) cells, one of the stem/progenitor cells in lung 2, in an ECF‐type solution over an ICF‐type solution 15. Based on these data, we hypothesized that organ‐preservation solutions, particularly ECF‐type solutions, could be used to preserve the viability of tissue‐resident stem cells. In this study, we developed a new preservation solution based on the ECF‐type organ‐preservation solution and evaluated its capacity to preserve the viability of tissue‐resident stem/progenitor cells.

## Materials and Methods

### Solutions

Our ECF‐type solution was prepared in collaboration with Cell Science Technology (Sendai, Japan). This ECF‐type solution is based on the organ‐preservation solution developed for lung transplantation 12. Because many potential solutions can be used for tissue preservation, we chose the solutions to be compared according to the following criteria: (a) the solution has been previously used for tissue preservation and/or transportation for experimental purpose; (b) the solution is chemically defined; and (c) the solution does not contain additives that can affect the stem/progenitor characteristics such as recombinant growth factors and animal serum. According to these criteria, we chose Ca^2+^‐ and Mg^2+^‐free PBS (Invitrogen, Carlsbad, CA, USA) and high‐glucose Dulbecco’s modified Eagle’s medium (DMEM; Invitrogen) as control ECF‐type solutions because they are both often used for the transportation of human tissues 16, 17, 18, 19. Further, we used an ICF‐type preservation solution (ICF, Euro‐Collins solution; I’rom Pharmaceutical Co., Tokyo, Japan) that does not contain additives such as growth factors or corticosteroids as a control for organ‐preservation‐type solutions 10. The compositions of these solutions are listed in Table 1.

**Table 1:** Composition of preservation solutions.

Components	ICF	ECF	PBS	DMEM
Na^+^ (mm)	10	141	157	155
K^+^ (mm)	115	26	1	5
Cl^−^ (mm)	15	103	155	117
Ca^2+^ (mm)	(−)	(−)	(−)	2
Mg^2+^ (mm)	(−)	(−)	(−)	1
${\text{HCO}}_{3}^{-}$ (mm)	10	(−)	(−)	44
$H_{2}{\text{PO}}_{4}^{-}$ (mm)	15	5	1	1
${\text{HPO}}_{4}^{2-}$ (mm)	42.5	60	3	(−)
Dextran 40 (g·L^−1^)	(−)	20	(−)	(−)
Glucose (g·L^−1^)	35.7	10	(−)	45
Amino acids (mm)	(−)	(−)	(−)	10.7^a^
Vitamins (mm)	(−)	(−)	(−)	0.2^b^

a Containing glycine, l‐arginine hydrochloride, l‐cystine, l‐glutamine, l‐histidine hydrochloride, l‐isoleucine, l‐leucine, l‐lysine hydrochloride, l‐methionine, l‐phenylalanine, l‐serine, l‐threonine, l‐tryptophan, l‐tyrosine disodium salt dehydrate and l‐valine.

b Containing choline chloride, d‐calcium pantothenate, folic acid, niacinamide, pyridoxine hydrochloride, riboflavin, thiamine hydrochloride and i‐inositol.

### Animal studies

Male C57BL/6 mice (CLEA Japan, Inc., Tokyo, Japan), 7–10 weeks old, were maintained in the animal facilities of the Tohoku University School of Medicine under specific pathogen‐free conditions. Animal experiments were conducted with approval from the Tohoku University Review Board.

#### Preservation protocol

Mice were euthanized by an overdose of halothane. After thoracotomy, lungs were perfused with 8 mL of each of the preservation solutions. Heart‐lung blocks (2 g of each) were isolated and stored at 4 °C for 72 h in 30 mL of the same solution as that used for lung perfusion.

#### Preparation of mouse lung single‐cell suspension

After 4 °C preservation, lungs were enzymatically treated, and single‐cell suspensions were prepared as previously described with minor modifications 20, 21. In brief, the lungs were incubated in a 37 °C shaking incubator for 45 min in 10 mL of Dispase (2 U·mL^−1^; Roche Diagnostics, Indianapolis, IN, USA), 1 mL of DNase (0.1 mg·mL^−1^; Sigma‐Aldrich, St. Louis, MO, USA) and 1 mL of collagenase/Dispase (2 µg·mL^−1^; Roche Diagnostics). The lungs were then minced and incubated for 10 min. The cell suspension was filtered using a 40‐µm filter (BD Biosciences, San Jose, CA, USA).

#### Cell death analysis by flow cytometry

Lung cells were labeled with an allophycocyanin‐conjugated anti‐(mouse stem cell antigen‐1) (Sca‐1) IgG (BD Pharmingen, San Jose, CA, USA), Annexin V and propidium iodide (PI; Annexin V‐FLUOS Staining Kit; Roche Diagnostics), and analyzed using a FACSCalibur (BD Biosciences).

#### Isolation and culturing of mouse Sca‐1^+^ lung cells

Sca‐1^+^ lung stem cells were isolated as described previously 20 using an AutoMACS system (Miltenyi Biotec, Bergisch Gladbach, Germany). Hematopoietic cells were depleted using mouse anti‐cluster of differentiation 45 (CD45) microbeads (Miltenyi Biotec). Sca‐1^+^/CD45^−^ lung cells were then selected using a fluorescein isothiocyanate (FITC)‐conjugated mouse anti‐Sca‐1 IgG and anti‐FITC microbeads (Miltenyi Biotec). The Sca‐1^+^/CD45^−^ lung cells were plated into six‐well plates with DMEM and 10% FBS (GIBCO, Carlsbad, CA, USA) at a density of 2 × 10^4^ cells·cm^−2^ on mitotically inactivated mouse embryonic fibroblasts (MEFs).

#### Evaluation of mouse lung stem cell properties

The number of colonies per well on day 7 was counted under an IX71 inverted microscope (Olympus, Tokyo, Japan). Fluorescence‐activated cell sorting (FACS) analysis was performed using antibodies against Sca‐1, CD45, CD31, CD34, CD90 and CD44 (all purchased from BD Pharmingen). Another aliquot of expanded cells was seeded at a density of 1 × 10^5^ cells·mL^−1^ in Matrigel (BD Bioscience) and cultured for 14 days, as previously described 20.

#### Immunofluorescence of mouse Sca‐1^+^ cells

Mouse lung cells were fixed, blocked and permeabilized with BD Cytofix/Cytoperm Kit, according to the manufacturer’s instructions. Cells were then incubated with goat anti‐mouse pro‐surfactant protein C) (proSP‐C; Santa Cruz Biotechnology, Dallas, TX, USA) or rat anti‐(mouse CD31) (BD Pharmingen) IgG at 4 °C overnight and then incubated for 1 h with Alexa Fluor 546 donkey anti‐(goat IgG) or Alexa Fluor 546 goat anti‐(rat IgG) (Molecular Probes, Carlsbad, CA, USA), respectively.

### Human study

#### Handling and preservation of human lung specimens

The handling and preservation of human lung specimens conformed to the guidelines set by the Declaration of Helsinki, and the study protocol was approved by the Ethics Committee at Tohoku University School of Medicine, Sendai, Japan, and Japanese Red Cross Ishinomaki Hospital, Ishinomaki, Japan. All patients provided their written informed consent. Human lung tissues were derived from surgical specimens of patients who underwent lobectomy for lung cancer at the Tohoku University Hospital and Japanese Red Cross Ishinomaki Hospital. The patient characteristics are described in Tables 2 and 3. Lung tissue samples at least 10 cm away from the tumors were used. Three grams of each human lung specimen was placed into 50‐mL conical tubes with 30 mL of ICF‐type or ECF‐type solution and then stored at 4 °C.

**Table 2:** Clinical characteristics of patients from whom lung stem/progenitor cell cultures were created.

Characteristics	Fresh	ECF preservation	ICF preservation
*n*	3	4	3
Age, years (minimum to maximum)	75 (70–78)	70 (59–78)	0.42
Male/Female	1/2	1/3	1/2
Smoker	1	1	1
FEV1.0/FVC (± SD)	65.5 (± 6.5)	76.1 (± 16.4)	72.1 (± 17.0)

FEV1.0, Forced Expiratory Volume in 1 s; FVC, Forced Vital Capacity.

**Table 3:** Clinical characteristics of patients from whom samples were taken for ATII cell isolation.

Characteristics	Days of preservation			
	0–2	3–5	6–8	9–11
*n*	9	14	7	8
Age, years (minimum to maximum)	73.0 (59–86)	74.9 (64–85)	69.9 (62–81)	69.5 (55–79)
Smoker	3	4	5	4
Male/Female	6/3	10/4	4/3	6/2
FEV1.0/FVC (± SD)	67.2 (± 12.5)	70.5 (± 10.7)	72.9 (± 11.3)	75.7 (± 14.4)
%FEV1.0 (± SD)	94.73 (± 29.3)	98.5 (± 19.6)	94.0 (± 14.2)	96.6 (± 16.6)

FEV1.0, Forced Expiratory Volume in 1 s; FVC, Forced Vital Capacity.

#### Culture and preservation of the human cell line

A549 lung epithelial cell line was cultured in DMEM supplemented by 10% of FBS (GIBCO). A172 glioma cell line was cultured in RPMI 1640 supplemented with 10% of FBS. Cells were collected and resuspended in either ECF‐type solution or ICF‐type solution at a density of 2 × 10^6^ cells·mL^−1^ and stored at 4 °C for 24 h. Then cells were subcultured in 24‐well culture plates at a density of 1 × 10^4^ cells·well^−1^. Cell number was counted on days 3, 5, 7 and 10 after preservation.

#### Preparation of single‐cell suspension and culturing of human lung cells

Human lung cell suspensions were prepared as previously described 3, 22. CD45^+^ cells were depleted using anti‐human CD45 microbeads and the AutoMACS system (Miltenyi Biotech). CD45^−^ human lung cells were then plated into six‐well plates with DMEM (Invitrogen) and 10% FBS (GIBCO) at a density of 2.5 × 10^4^ cells·cm^−2^ on MEFs.

#### Flow cytometry and sorting of lung cells

Phycoerythrin (PE)‐conjugated anti‐(human epithelial cell adhesion molecule) (EpCAM) IgG (clone 1B7; eBioscience, San Diego, CA, USA), Alexa Fluor 647–conjugated anti‐(human T1a) IgG (clone NC‐08; BioLegend, San Diego, CA, USA) and 7‐amino actinomycin D (eBioscience) were used for sorting viable ATII cells. The gating strategy on the FACSAria II Cell Sorter and FACSDiva (BD Biosciences) were as previously reported 23.

#### Evaluation of human lung stem cell properties

Cell surface markers were analyzed by FACSCantoII with antibodies against CD45(FITC), CD31(FITC), CD73(PE), CD90(FITC) and CD105(PE) (all purchased from BD Pharmingen). The mesenchymal cell differentiation capacity of the cells was confirmed using a Human Mesenchymal Stem Cell Functional Identification Kit (#SC006; R&D Systems, Minneapolis, MN, USA). In brief, for adipogenic differentiation, cells were cultured in Alpha Minimum Essential Medium supplemented with 10% FBS, hydrocortisone, isobutylmethylxanthine and indomethacin. Adipocytic differentiation was confirmed using Oil Red O staining, according to standard laboratory protocols, and immunofluorescence using an anti‐(fatty acid–binding protein 4) (FABP‐4) IgG. Osteogenic differentiation was performed by culturing cells in Alpha Minimum Essential Medium containing 10% FBS, dexamethasone, ascorbate‐phosphate, proline, pyruvate and recombinant transforming growth factor‐β3. Osteogenic differentiation was confirmed by Alizarin Red staining, according to standard laboratory protocols, and immunofluorescence using a human Osteocalcin antibody, according to the manufacturer’s instructions.

#### Total RNA Isolation and RT‐PCR

Total RNA was extracted using an RNeasy Mini Kit (QIAGEN, Tokyo, Japan). cDNA was synthesized with QuantiTect Reverse Transcription Kit (QIAGEN). The primer sequences are shown in Table 4.

**Table 4:** Sequences of primers and PCR conditions. KRT19, cytokeratin 19; α‐SMA, α‐smooth muscle actin.

Gene	Direction	Sequence	Annealing temperature	Cycle number	Size
*SP‐B*	Forward	5′‐TGGGAGCCGATGACCTATG‐3′	62	40	70
	Reverse	5′‐GCCTCCTTGGCCATCTTGT‐3′			
*SP‐C*	Forward	5′‐ATCGGCTCCACTGGCCTCGT‐3′	62	40	301
	Reverse	5′‐AGTAGAGCGGCACCTCGCCA‐3′			
*KRT19*	Forward	5′‐TTTGAGACGGAACAGGCTCT‐3′	60	40	211
	Reverse	5′‐AATCCACCTCCACACTGACC‐3′			
*Vimentin*	Forward	5′‐CCCTCACCTGTGAAGTGGAT‐3′	60	35	241
	Reverse	5′‐TCCAGCAGCTTCCTGTAGGT‐3′			
*α‐SMA*	Forward	5′‐TTCAATGTCCCAGCCATGTA‐3′	60	35	222
	Reverse	5′‐GAAGGAATAGCCACGCTCAG‐3′			
*β‐Actin*	Forward	5′‐GCTCGTCGTCGACAACGGCTC‐3′	55	30	353
	Reverse	5′‐CAAACATGATCTGGGTCATCTTCTC‐3′			

### Statistics

Data are expressed as the mean ± SD. Statistical analysis was performed with graphpad prism software (GraphPad Software, La Jolla, CA, USA). Student’s *t* test, one‐way ANOVA, followed by Tukey‐Kramer multiple comparison tests or two‐way ANOVAs were performed as appropriate.

## Results

### Mouse study

#### Impaired viability of Sca‐1^+^ lung cells after DMEM and PBS preservation

**Figure 1:**
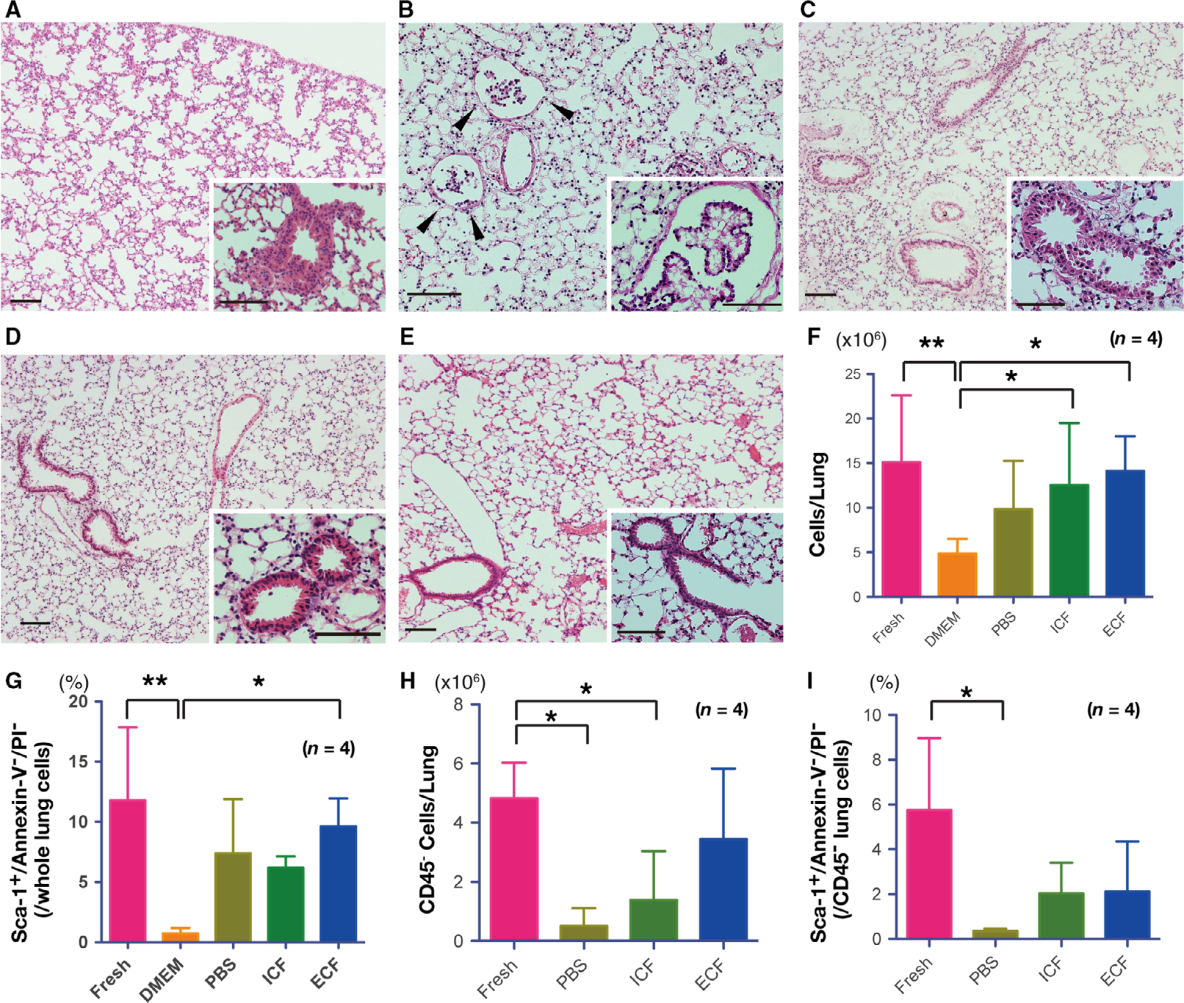
Viability of mouse lung stem cells after preservation. Hematoxylin and eosin–stained images after preservation for 3 days in DMEM (A), PBS (B), ICF‐type solution (C) and ECF‐type solution (D). (E) Freshly fixed lung. Scale bars indicate 100 µm (A–E). (F) Total lung cell counts from fresh lungs and preserved lungs. (G) Percentage of Annexin V^−^/PI^−^/Sca‐1^+^ cells among whole lung cells. (H) CD45^−^ cell counts from fresh lungs and preserved lungs. (I) Percentage of Annexin V^−^/PI^−^/Sca‐1^+^ cells among CD45^−^ lung cells. (F–I) Four biological repeats; error bars indicate SD. Statistical differences were tested using one‐way ANOVA with Tukey’s multiple comparison test. *P < 0.05 and **P < 0.01 indicate significant difference.

In DMEM‐ or PBS‐preserved lungs, we observed marked epithelial cell swelling or detachment, particularly in the bronchioalveolar junctions (Fig. 1A,B), which are considered to be the stem cell niches in the mouse lung 2. By comparison with the fresh lungs (Fig. 1E), the lung structure was generally intact in the ICF‐ or ECF‐preserved lungs (Fig. 1C,D). In addition, total cell counts after 72 h of preservation were significantly lower in the DMEM‐preserved lungs (Fig. 1F).

The Sca‐1^+^ cell population comprises resident stem cells in the mouse lung 20, 23, 24. Therefore, the percentage of Annexin V^−^/PI^−^/Sca‐1^+^ cells among whole lung cells would provide an estimate of the viability of stem/progenitor cells. We found that the percentage of viable Sca‐1^+^ cells was significantly lower in DMEM‐preserved lungs as compared with the fresh control and ECF‐preserved lungs (Fig. 1G). The incidence of the Annexin V^−^/PI^−^ condition among Sca‐1^+^ cells was also lower in the DMEM‐preserved lungs (8.4% ± 4.7%) as compared with the fresh (43.0% ± 16.0%), PBS‐preserved (33.6% ± 10.6%), ICF‐preserved (19.6% ± 5.9%) and ECF‐preserved lungs (31.1% ± 5.1%), with statistical significance between DMEM‐preserved and fresh, DMEM‐preserved and PBS‐preserved, and DMEM‐preserved and ECF‐preserved conditions. These results indicate that DMEM is not suitable for stem cell preservation. Therefore, we excluded DMEM as a preservation solution in subsequent experiments.

After depleting CD45^+^ cells, we found that the number of viable cells counted by trypan blue exclusion was significantly lower in PBS‐preserved and ICF‐preserved lungs than in the fresh control; however, there were no significant differences in the number of CD45^−^ cells between the fresh and ECF‐preserved samples (Fig. 1H). The percentage of Annexin V^−^/PI^−^/Sca‐1^+^ cells among total CD45^−^ lung cells was significantly lower in the PBS‐preserved lungs than in the fresh control lungs (Fig. 1I). The estimated viable Sca‐1^+^/CD45^−^ cell numbers in the PBS‐preserved lung samples were always less than 2 × 10^3^ (four repeats). Consequently, because we could not isolate a sufficient number of viable Sca‐1^+^/CD45^−^ cells, we excluded PBS from subsequent experiments.

#### Viability of colony‐forming Sca‐1^+^ cells was efficiently maintained in ECF‐type solution

**Figure 2:**
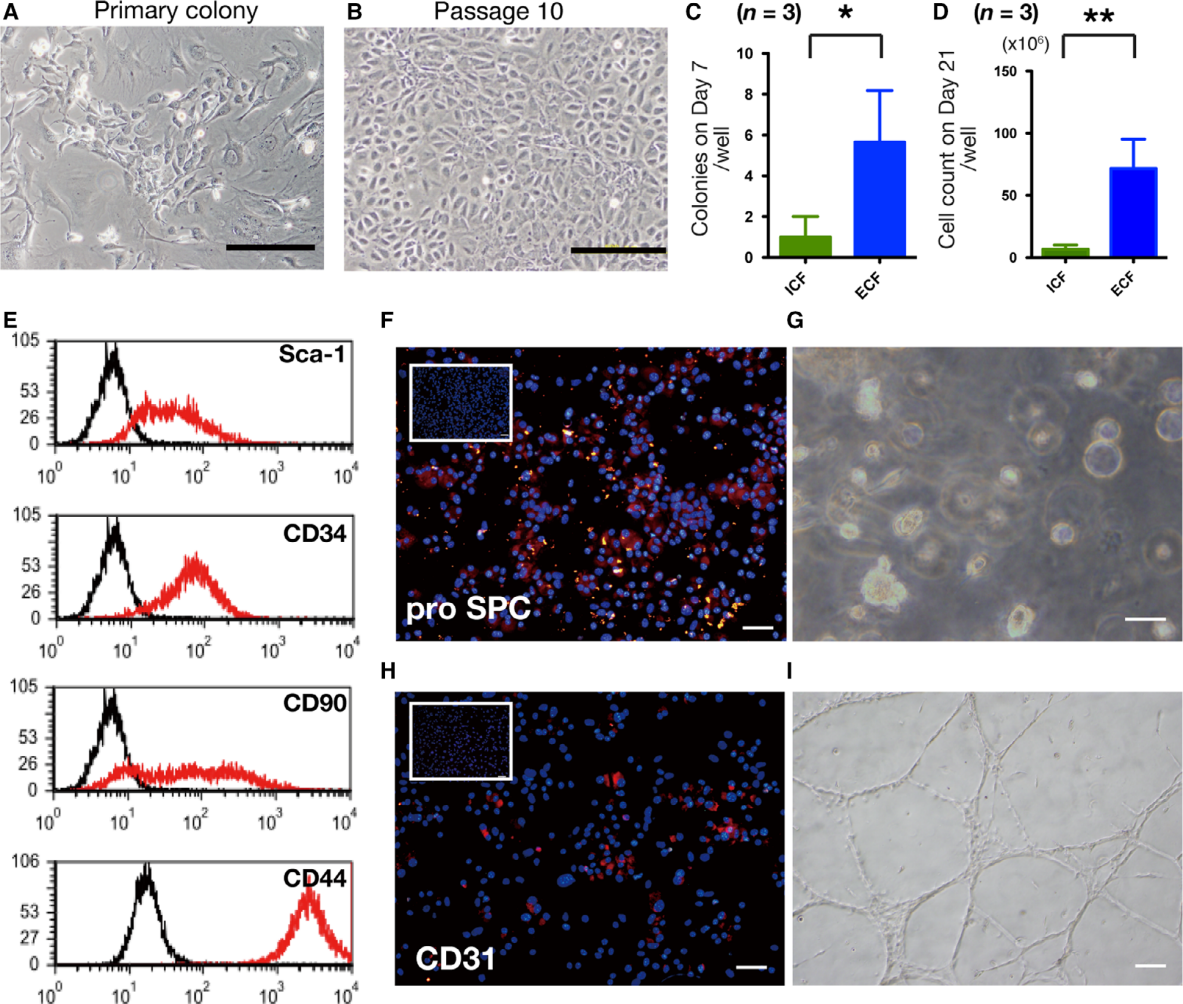
Expansion, characterization and differentiation capacity of ECF‐preserved Sca‐1^+^ lung cells. (A) Isolated Sca‐1^+^ cells developed colonies on MEF feeder cells. (B) Expanded cells from ECF‐preserved Sca‐1^+^ cells at passage 10. (C) Colony counts of cells from ICF‐preserved lungs and ECF‐preserved lungs after 7 days of culturing. (D) Cell counts of cells from ICF‐preserved lungs and ECF‐preserved lungs on day 21 of culturing. (E) Representative flow cytometry analysis of the expanded cells (passage 10) derived from ECF‐preserved lungs. (F–I) After 14 days on Matrigel, the expression of proSP‐C and CD31 was upregulated (F, H) as compared with baseline expression (inset, white boxes; expanded cells at passage 10). Sca‐1^+^ cells demonstrated alveolar‐like sphere formation (G) and tubelike structures (I). Scale bars indicate 50 µm. (C, D) Three biological repeats; error bars indicate SD. Statistical differences were tested using Student’s *t*‐test. *P < 0.05 and **P < 0.01 indicates significant difference.

For the Sca‐1^+^/CD45^−^ population, the colony counts on day 7 and the cell counts on day 21 were significantly higher in the ECF preservation solution than in the ICF preservation solution (Fig. 2C,D). Notably, ICF‐preserved Sca‐1^+^ cells formed colonies on feeder cells; however, they stopped growing within 30 days in three independent experiments. In contrast, cultured Sca‐1^+^ cells derived from ECF‐preserved lungs were successfully passaged until passage 10 (three repeats; Fig. 2A,B). These cells expressed stem cell markers at various levels, including Sca‐1 (67–94%), CD34 (10–89%), CD90 (68–99%) and CD44 (99%; Fig. 2E), but did not express lineage markers, such as CD31 or CD45 (data not shown), as per previous reports 20, 23. After 14‐day differentiation culture on Matrigel, we confirmed the expression of proSP‐C and CD31 by immunofluorescence (Fig. 2F,H). In addition, Sca‐1^+^ cells from ECF‐preserved lungs formed alveolar‐like sphere formation and tubelike structures (Fig. 2G,I). These observations indicated that Sca‐1^+^ lung cells derived from ECF‐preserved lungs maintained their capacity to differentiate. This multilineage differentiation capacity of the Sca‐1^+^/CD45^−^ cells was compatible with the multipotent lung stem cells previously reported 20.

### Human study

#### Viability and growth potential of lung cells after preservation in organ‐preservation solution

**Figure 3:**
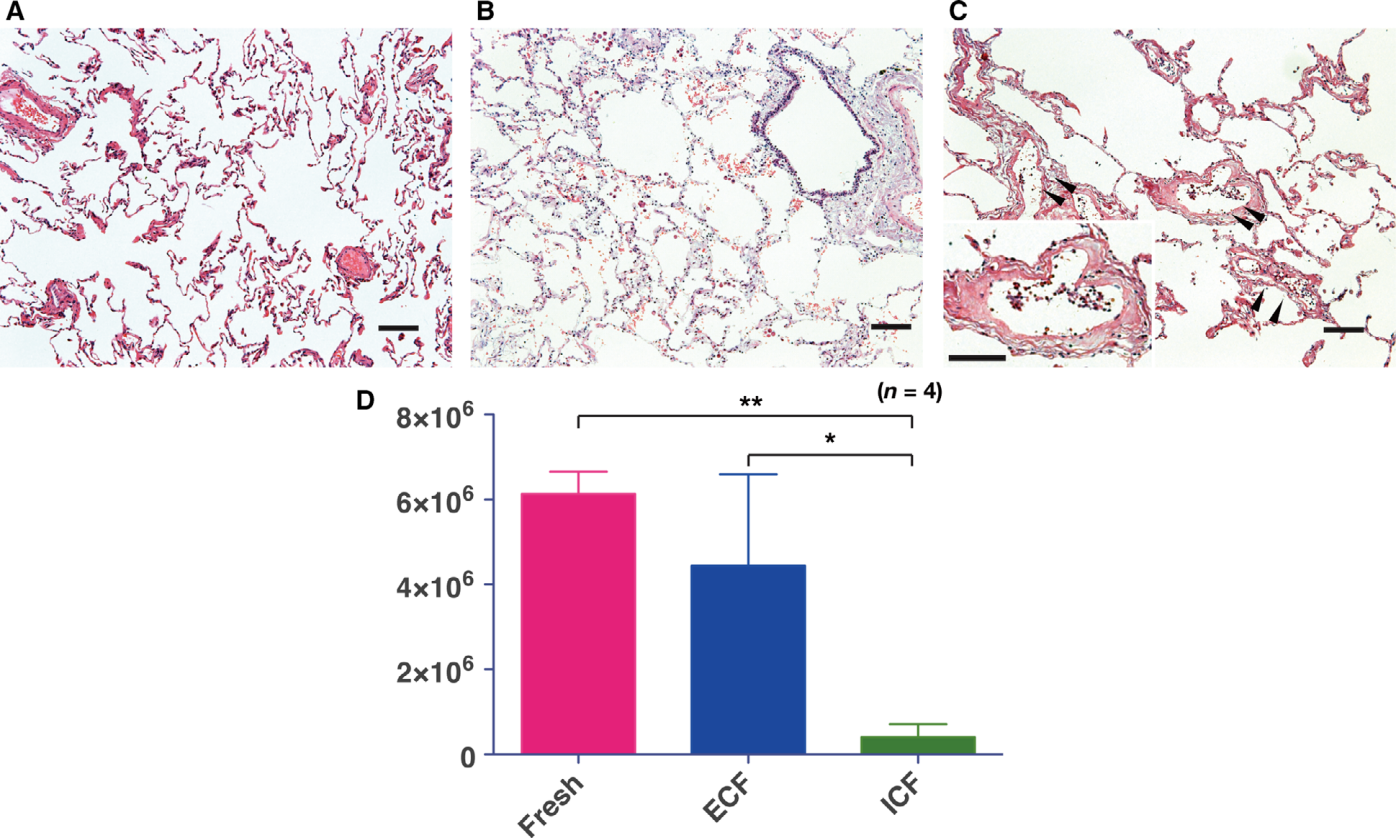
Viability of lung cells after 7‐day preservation in organ preservation solutions. Hematoxylin and eosin staining of fresh (A), ECF‐preserved (B) and ICF‐preserved lung tissue (C). Cell detachment was observed in ICF‐preserved lung tissue (C, arrowheads, inset). Viable lung cell counts from 1 g of lung tissue after 7‐day preservation (D). Scale bars indicate 100 µm (A–C). (D) Four biological repeats, error bars indicate SD. Statistical difference was tested using one‐way ANOVA with Tukey’s multiple comparison test. *P < 0.05 and **P < 0.01 indicate significant difference.

There was no apparent deterioration in lung alveolar structure after preservation for 7 days in ECF‐type solution (Fig. 3A,B); however, we observed cell detachment in small airways in ICF‐preserved lungs (Fig. 3C). The number of viable CD45^−^ lung cells after 7 days in ECF‐preservation solution was 4.4 × 10^6^ ± 2.1 × 10^6^ per gram of lung tissue and after 7 days in ICF‐preservation solution was 4.0 × 10^5^ per gram of lung tissue, whereas that from fresh lung tissue was 6.1 × 10^6^ ± 0.5 × 10^6^ per gram of lung tissue. CD45^−^ cell counts from 1 g of lung tissue after 7‐day preservation were significantly lower in ICF‐preserved lungs (Fig. 3D). We then examined the influence of preservation solutions to the growth potential of immortalized cell lines A549 (lung adenocarcinoma) and A172 (glioma). Interestingly, the growth of A549 cells was significantly lower after ICF preservation than the growth after ECF preservation (Fig. S1A), which was also confirmed using the A172 glioma cell line (Fig. S1B).

#### Viability of colony‐forming human lung cells in ECF‐type solution for 7 days

**Figure 4:**
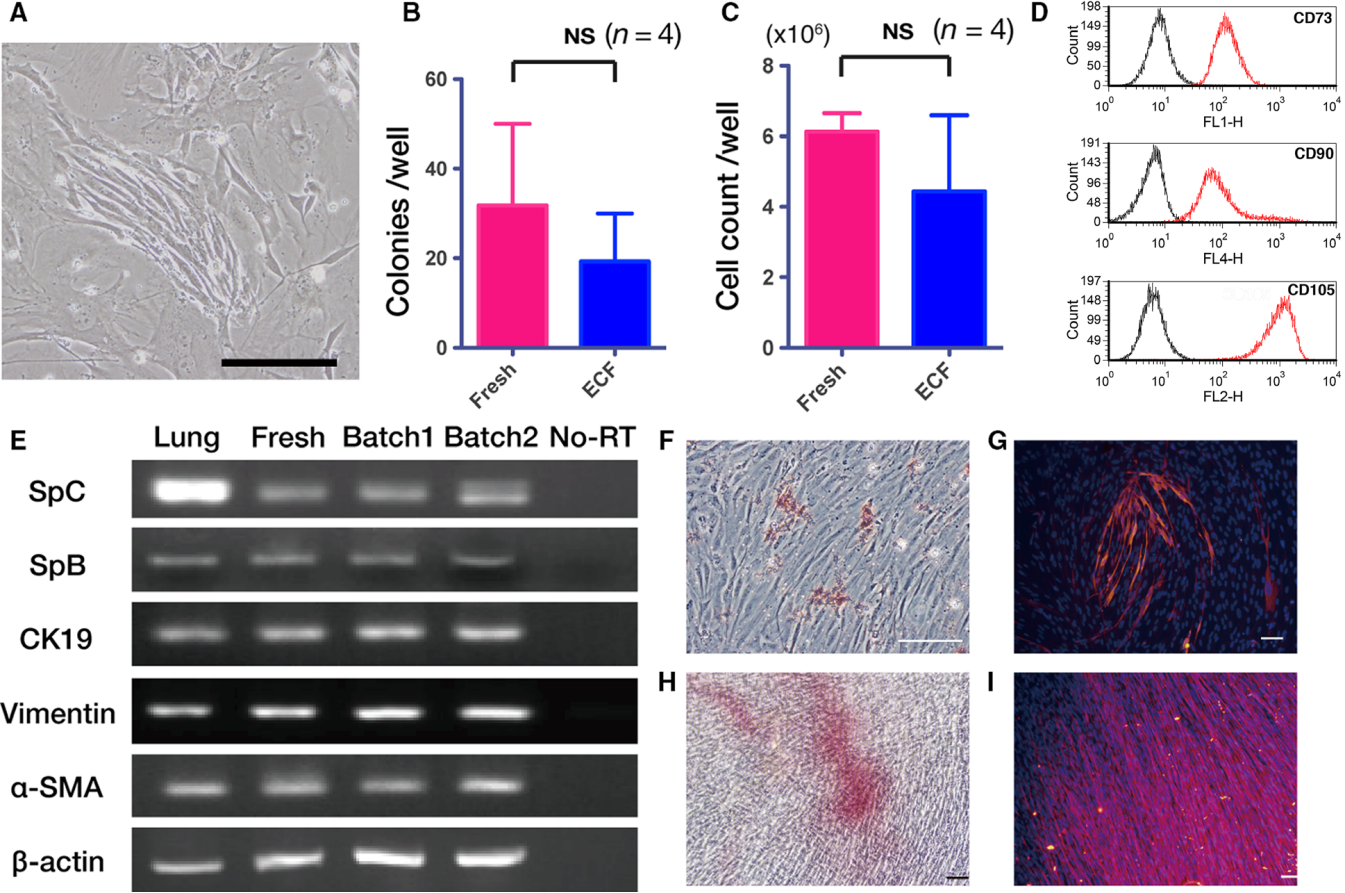
Colony formation and differentiation capacity of ECF‐preserved human lung progenitor cells. (A) A representative image of the colony derived from ECF‐preserved human lungs. (B) Colony counts of human lung cells after 10 days of culturing. (C) Cell counts after 21 days of culturing. (D, E) Expanded cells expressed alveolar epithelial type II progenitor cell markers CD73, CD90 and CD105 (D), and proSP‐C, SP‐B and cytokeratin 19 (CK19) (E). The expanded cells also expressed mesenchymal stem or stromal cell markers, vimentin and α‐smooth muscle actin (SMA) (E). (F, G) After 21 days of adipogenic culture, lipid droplets were observed by Oil Red O staining (F), and FABP‐4^+^ cell staining was also detected (G). After 21 days, osteogenic differentiation was confirmed by Alizarin Red S staining (H) and osteocalcin expression (I). Scale bars indicate 50 µm (A, F–I). (B, C) Four biological repeats; error bars indicate SD. Statistical differences were tested using Student’s *t*‐test. Batch 1 and batch 2, two biological replicates of lung stem cells from different lung specimens after ECF preservation; Fresh, freshly isolated lung cells; Lung, whole lung tissue lysate; No‐RT, no reverse transcriptase control; NS, not significant.

We next evaluated colony formation of human lung stem cells isolated from preserved human lung tissues 25. CD45^−^ cells from 7‐day preservation in ICF solution did not form a colony on feeder cells in three repeats. The isolated cells from ECF‐preserved lungs developed colonies within 10 days (Fig. 4A). The colony count for lung cells preserved in ECF after 10 days of culturing was 60% of that of the fresh control; nevertheless, there was no significant difference between these two groups (Fig. 4B). The cell count for lung cells after 21 days of culturing was also comparable between ECF and fresh conditions (Fig. 4C).

These colony‐forming cells were both morphologically and phenotypically identical to mesenchymal stem or stromal cells reported previously 3, 22. The passaged cells expressed CD73, CD90 and CD105 (Fig. 4D) but did not express CD45, CD31 or CD34 (data not shown). Importantly, after 21 days of primary culture on feeder cells, the expanded cells expressed epithelial genes such as proSP‐C, SP‐B and cytokeratin 19 (Fig. 4E), which are characteristic of adult epithelial type II progenitor cells in human lungs 22. Adipocytic differentiation of the cells was confirmed by Oil Red O staining and FABP‐4 expression (Fig. 4F,G). Osteogenic differentiation of the cells was confirmed by Alizarin Red staining and osteocalcin expression (Fig. 4H,I).

#### Viability of human ATII cells in the ECF‐preservation solution

**Figure 5:**
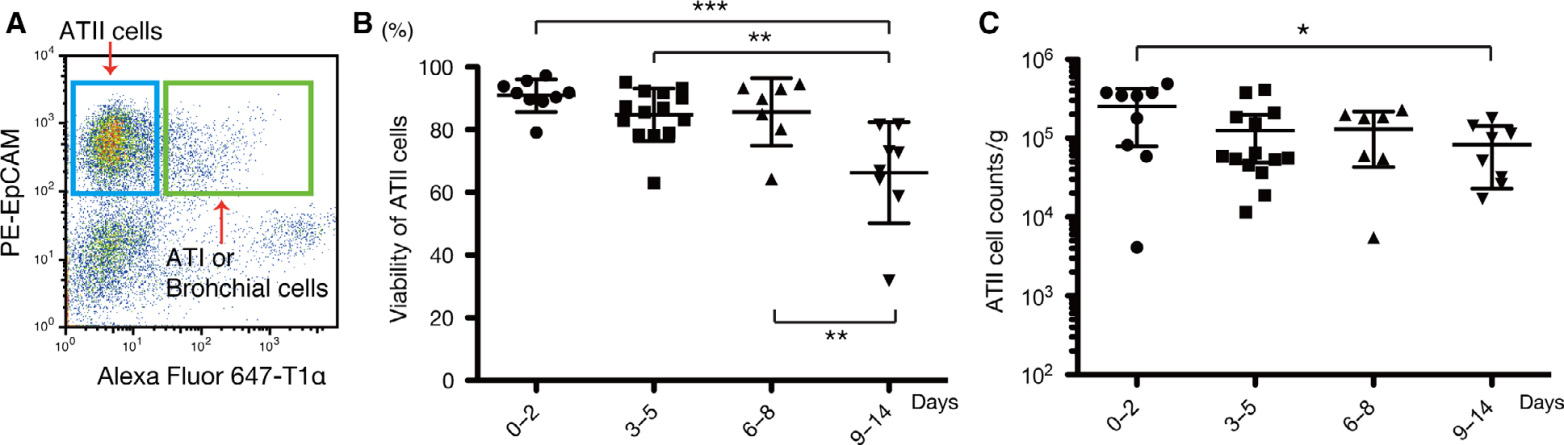
Viability of ATII cells after preservation in ECF‐type solution. (A) Gating strategy to isolate ATII cells. ATII cells are enriched in the EpCAM^high^/T1α^−^ subset, whereas the EpCAM^low^/T1α^+^ subset contains mainly ATI cells and bronchial epithelial cells. (B) Viabilities of EpCAM^high^/T1α^−^ cells derived from lung specimens that were preserved in ECF‐type solution for the indicated periods. (C) Number of viable EpCAM^high^/T1α^−^ cells derived from lung specimens that were preserved in ECF‐type solution for the indicated periods. *P < 0.05, **P < 0.01 and ***P < 0.001 indicate significant difference.

We finally evaluated the viability of ATII cells, which are considered to be one of the lung‐specific progenitor cells. The isolation of ATII cells was performed using the combination of antibodies as previously reported 25. An ATII‐enriched population was in the EpCAM^high^/T1α^−^ subset, whereas the EpCAM^low^/T1α^+^ population contained a mixture of ATI cells and other bronchial cells (Fig. 5A) 25, 26, 27. The isolated EpCAM^high^/T1α^−^ population, which should have contained no less than 95% of ATII cells according to the previous report 25, maintained high viability until 8 days of preservation in ECF‐type solution (Fig. 5B). The yields of ATII‐enriched cells per gram of lung tissue gradually decreased with preservation time, yet there was no statistically significant difference until day 8 (Fig. 5C).

## Discussion

Our results demonstrate that the viability of tissue‐resident stem/progenitor cells was significantly improved when using an organ‐preservation solution. Viability was particularly evident using the newly developed and chemically defined ECF‐type solution, which potentially could maintain human lung stem/progenitor cell viability at 4 °C for 1 week. It is believed that stem cell viability will significantly decrease after 48 h postmortem 8. In previous investigations, therefore, fresh clinical samples were empirically stored in a generic buffer or media without a quantitative evaluation of the tissue and cell viability after preservation as compared with samples that are otherwise processed immediately after collection 16, 17, 18. Our results show how the viability of tissue‐resident stem/progenitor cells can be substantially influenced by the composition of the solution used for storage. The viability of Sca‐1^+^ lung cells after preservation in DMEM was significantly lower than the viability of Sca‐1^+^ cells after preservation in the other solutions. PBS showed comparable preservation capacity to ICF‐ or ECF‐type solution in terms of preserving the viability of Sca‐1^+^ whole lung cells (Fig. 1D); however, the viability of Sca‐1^+^ cells after PBS preservation was significantly lower than the viability of Sca‐1^+^ cells after ICF or ECF preservation when CD45^+^ hematopoietic cells were depleted, suggesting that the viable Sca‐1^+^ cells after PBS preservation were mainly hematopoietic‐derived cells (Fig. 1H,I).

The reason the ECF‐type solution yielded better results than the other solutions remains to be determined. Previous reports might partially explain our results: (a) solutions with high potassium concentrations induce an influx of calcium, which results in mitochondrial calcium overload and cellular apoptosis 28; (b) cell‐impermeant molecules, such as dextran, can reduce cell swelling by maintaining the cell membrane integrity 10, 28; and (c) adequate buffering prevents cells from severe acidosis during hypothermic preservation 29. Among the solutions tested in this study, only the ECF‐type solution comprised all of these traits: glucose, low‐molecular‐weight dextran and a high amount of phosphate buffer. These components might have influenced stem cell viability during preservation. For example, the use of bicarbonate, which requires continuous supply of carbon dioxide for its buffering ability, might explain why DMEM and Euro‐Collins solution were less capable of maintaining the cell viability during the refrigerated storage.

One of the limitations of this study is the limited types of solution that were compared. There exist more than 100 types of preservation solutions in the field of organ transplantation 30, and we included only Euro‐Collins solution in this study and did not compare other widely used preservation solutions such as University of Wisconsin, histidine‐tryptophan‐ketoglutarate, Celsior or Perfadex 31. These preservation solutions, which have demonstrated the ability to facilitate the organ viability and function during refrigerated storage, also can be beneficial during tissue transport for the isolation and culture of adult cells; however, considering the potential impairment of cell proliferation in high extracellular potassium concentration conditions substantiated by our results and previous investigations 32, 33, 34, care should be taken when using ICF‐type solution for tissue preservation. Another limitation is that the tissue stem cell function after preservation was examined only by comparing the colony formation ability of lung mesenchymal stem cells whose origin and function are still controversial 18, 19, 22, 35, 36. In addition, the purity of ATII cells, which were isolated according to the expression pattern of EpCAM and T1α, was not confirmed by SpC immunoexpression, and neither the proliferation nor differentiation of ATII cells was examined in a series of lung tissue preservation (Fig. 5). Therefore, this study cannot substantiate that the isolated lung stem/progenitor cells after preservation in the ECF‐type solution are in the same condition as they are* in vivo. *However, the improvement of cellular viability during tissue transportation that can be achieved using the newly developed ECF‐type solution will help researchers widen the availability of precious human specimens for investigation and clinical application.

This study focused on tissue‐resident stem cells in the lung; therefore, the applicability of the ECF solution for the preservation of other types of stem cells should be confirmed independently. However, considering that previous investigations have demonstrated that dextran‐containing ECF‐type solutions protect the viability of other organs 10, 13, the viability of tissue‐resident stem/progenitor cells of other organs would also likely be improved by using an organ‐preservation solution.

## Conclusions

Viability of tissue‐resident stem/progenitor cells after extended refrigerated storage can be improved using ECF‐type organ preservation solution.

## Conflict of interest

The authors declare no conflict of interest.

## Author contributions

TS and HK conceived and designed the project. TS, CO, NF, YT and SS acquired the data. TS, MY, TK, YO and HK analyzed and interpreted the data. TS and HK wrote the manuscript.

## Supporting information


**Fig S1.** The growth curve of the human cancer cell line after 24 h of 4 °C storage.

## Data Availability

All of the data used to support the findings of this study are included within the article and the supplementary material files.
