# Supplementary figures and images for: Improving the viability of tissue‐resident stem cells using an organ‐preservation solution
Source: FEBS Open Bio. 2019 Nov 18;9(12):2093–104. doi: 10.1002/2211-5463.12748 (PMC6886303; doi:10.1002/2211-5463.12748)

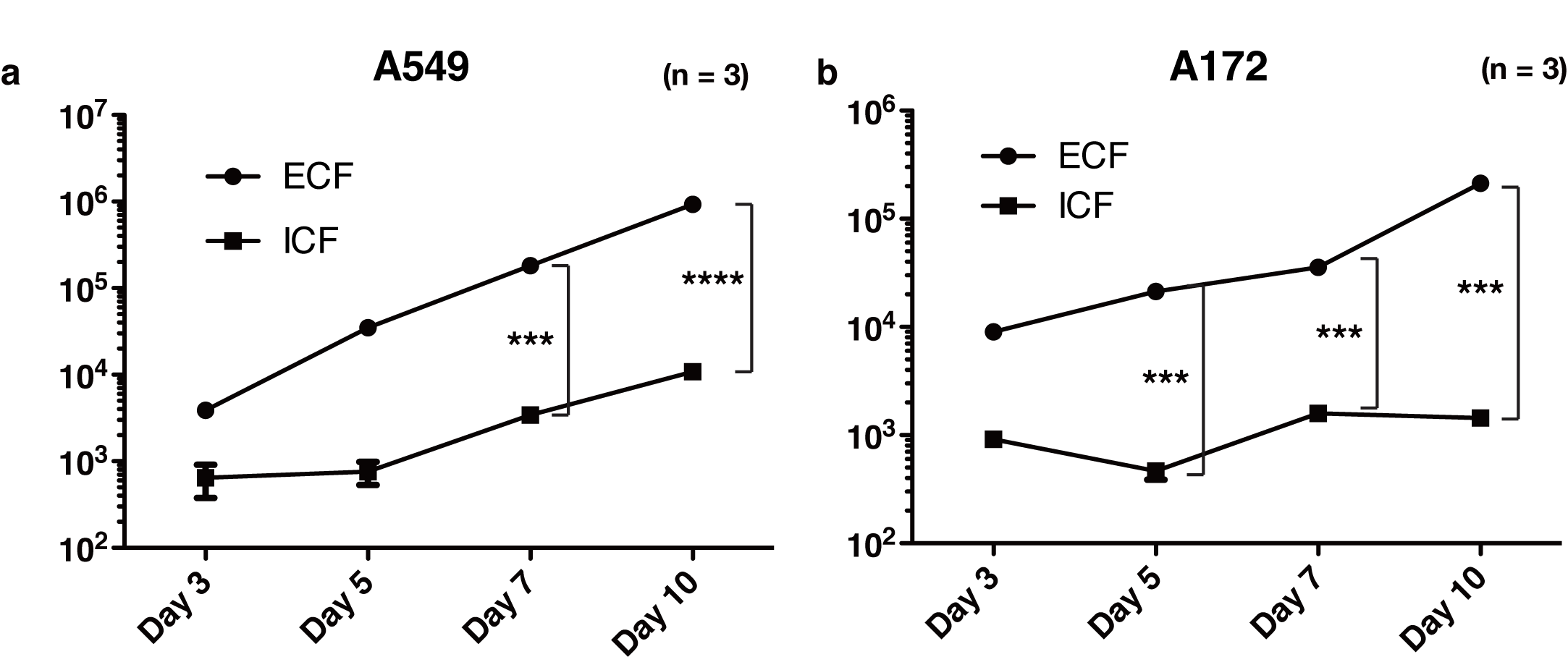

Supplement: Supplementary file 1 — Fig S1. The growth curve of the human cancer cell line after 24 h of 4 °C storage. [file FEB4-9-2093-s001.tif]
