# Supplementary material for: Improving the viability of tissue‐resident stem cells using an organ‐preservation solution
Source: FEBS Open Bio. 2019 Nov 18;9(12):2093–104. doi: 10.1002/2211-5463.12748 (PMC6886303; doi:10.1002/2211-5463.12748)
Supplement: Supplementary file 2 [file FEB4-9-2093-s002.docx]

Fig S1: Human cell line A549 and A172 were recovered, suspended in the preservation solutions for 24 hours and re-plated on cell culture plates. The cell counts were significantly higher after the preservation in ECF-type solution than after preservation in ICF-type solution in both A549 (a) and A172 (b). Three biological replicates, error bars indicate SD. 2-way ANOVA with Bonferroni multiple comparison test were used to test significant differences. ***, p<0.001. ****, p<0.0001.
